# Characteristics and outcomes of patients undergoing high-dose chemotherapy and autologous stem cell transplantation admitted to the intensive care unit: a single-center retrospective analysis

**DOI:** 10.1007/s00277-022-05028-x

**Published:** 2022-11-17

**Authors:** Jorge Garcia Borrega, Boris Böll, Matthias Kochanek, Jan-Hendrik Naendrup, Florian Simon, Noelle Sieg, Michael Hallek, Peter Borchmann, Udo Holtick, Alexander Shimabukuro-Vornhagen, Dennis A. Eichenauer, Jan-Michel Heger

**Affiliations:** First Department of Internal Medicine, Faculty of Medicine and University Hospital Cologne, Center for Integrated Oncology Aachen Bonn Cologne Dusseldorf, University of Cologne, Kerpener Str. 62, D-50937 Cologne, Germany

**Keywords:** Autologous stem cell transplantation, Intensive care unit, Mechanical ventilation, Prognosis

## Abstract

High-dose chemotherapy and autologous stem cell transplantation (ASCT) can be associated with adverse events necessitating treatment on the intensive care unit (ICU). Data focusing on patients admitted to the ICU during hospitalization for high-dose chemotherapy and ASCT are scarce. We thus conducted a single-center retrospective analysis comprising 79 individuals who had high-dose chemotherapy and ASCT between 2014 and 2020 and were admitted to the ICU between the initiation of conditioning therapy and day 30 after ASCT. The median age was 57 years (range: 20–82 years); 38% of patients were female. B-cell non-Hodgkin lymphoma (34%) and plasma cell disorders (28%) were the most common indications for high-dose chemotherapy and ASCT. Sepsis represented the major cause for ICU admission (68%). Twenty-nine percent of patients required mechanical ventilation (MV), 5% had renal replacement therapy, and 44% needed vasopressors. The ICU, hospital, 90-day, and 1-year survival rates were 77.2%, 77.2%, 72.2%, and 60.3%, respectively. Stable disease or disease progression prior to the initiation of high-dose chemotherapy (*p* = 0.0028) and MV (*p* < 0.0001) were associated with an impaired survival. A total of 36 patients died during observation. The most frequent causes of death were the underlying malignancy (44%) and sepsis (39%). Taken together, the present analysis indicates a favorable overall outcome for patients admitted to the ICU during hospitalization for high-dose chemotherapy and ASCT. Thus, this patient group should not be denied admission and treatment on the ICU.

**Supplementary information:**

The online version contains supplementary material available at 10.1007/s00277-022-05028-x.

## Introduction

High-dose chemotherapy followed by autologous stem cell transplantation (ASCT) is part of the initial therapy or salvage approaches in different hematologic malignancies and solid tumors [1–6]. Since high-dose chemotherapy and ASCT are associated with prolonged neutropenia and thrombocytopenia, patients undergoing this treatment modality are at risk for the development of infectious complications and other severe adverse events that can necessitate admission to the intensive care unit (ICU).

Data on characteristics and course of patients who had high-dose chemotherapy followed by ASCT and were admitted to the ICU are scarce [7–9]. This is especially true for individuals in whom ICU admission occurred during conditioning therapy or early after ASCT. We thus conducted a retrospective analysis comprising patients admitted to the ICU between the initiation of high-dose chemotherapy and day 30 after ASCT.

## Patients and methods

Patients aged ≥ 18 years who had received high-dose chemotherapy and ASCT at an academic tertiary care center (University Hospital Cologne) between January 1, 2014, and December 31, 2020, and had been admitted to the ICU between the initiation of high-dose chemotherapy and day 30 after ASCT were eligible for the present analysis. The institution’s ICU has of a total of 26 beds. High-flow nasal cannula oxygen therapy, renal replacement therapy (RRT), and vasopressor therapy can be conducted on all 26 beds, whereas the ability to conduct non-invasive ventilation therapy and mechanical ventilation (MV) is restricted to 14 beds.

Information on patient characteristics, laboratory parameters, the Hematopoietic Cell Transplantation-specific Comorbidity Index (HCT-CI) score at initiation of high-dose chemotherapy, treatment-related information, causes for ICU admission, the Sequential Organ Failure Assessment (SOFA) score at ICU admission, and procedures performed during the ICU stay were extracted from the patient charts [10, 11].

Numbers and proportions were indicated for dichotomous variables. Medians and ranges were calculated for continuous variables. Survival curves were obtained using the Kaplan–Meier method. Overall survival (OS) was defined as the time from ASCT until death from any cause and was censored at the date of last information for surviving patients. The influence of variables on OS was analyzed using the log-rank test (Mantel-Cox). Multivariable analysis including factors that were chosen according to their clinical relevance was performed using the Cox-regression method. Statistical significance was set to *p* < 0.05 (two-sided). The statistical analyses were performed using Microsoft Excel (version 16.45) and RStudio (version 2022.02.0) software for Mac.

## Results

### Baseline patient characteristics

Between January 1, 2014, and December 31, 2020, 738 patients underwent high-dose chemotherapy and ASCT at the University Hospital Cologne. Of these, 79 (10.7%) were admitted to the ICU between the initiation of high-dose chemotherapy and day 30 after ASCT and thus included in the present analysis. The median age was 57 years (range: 20–82 years). Females accounted for 30/79 cases (38%). B-cell non-Hodgkin lymphoma (B-NHL) and plasma cell disorders (PCD) represented the most common indications for high-dose chemotherapy and ASCT (B-NHL: 27/79 patients, 34.2%; PCD: 22/79 patients, 27.8%), whereas Hodgkin lymphoma (15/79 patients, 19%), T-cell non-Hodgkin lymphoma (9/79 patients, 11.4%), and solid tumors (6/79 patients, 7.6%) were less frequent indications. The median HCT-CI score at the initiation of high-dose chemotherapy was 3 (range: 0–9). Most patients had either complete remission (CR) or partial remission (PR) (CR: 24/79 patients, 30.8%; PR: 34/79 patients, 43%) prior to high-dose chemotherapy (Table 1).

**Table 1:** Characteristics of patients admitted to the ICU between the initiation of high-dose chemotherapy and day 30 after ASCT

	All patients		Survivors		Non-survivors	
		%		%		%
Total patients (n)	79		61	77.2	18	22.8
Age—median (range)	57 (20–82)		57 (20–82)		57 (26–75)	
Females (n)	30	38	26	42.6	4	22.2
Indication for HDCT and ASCT						
HL (n)	15	19	12	19.7	3	16.7
B-NHL* (n)	27	34.2	21	34.4	6	33.3
T-NHL (n)	9	11.4	7	11.5	2	11.1
PCD (n)	22	27.8	17	27.9	5	27.8
Solid tumor (n)	6	7.6	4	6.6	2	11.1
Initial diagnosis (n)	38	48.1	28	45.9	10	55.6
Relapsed/refractory disease (n)	41	51.9	33	54.1	8	44.4
Previous HDCT and ASCT	7	8.9	6	9.8	1	5.6
HCT-CI—median (range)	3 (0–9)		2 (0–9)		3 (0–8)	
HCT-CI > 2	41	51.9	30	49.2	11	61.1
Remission status before HDCT and ASCT						
CR (n)	24	30.8	20	32.8	4	22.2
PR (n)	34	43	28	45.9	6	33.3
SD (n)	11	13.9	7	11.5	4	22.2
PD (n)	10	12.7	6	9.8	4	22.2
Conditioning regimen						
BEAM (n)	33	41.8	27	44.3	6	33.3
Melphalan (n)	26	32.9	19	31.1	7	38.9
BCNU/thiotepa (n)	11	13.9	9	14.8	2	11.1
Busulfan/thiotepa (n)	3	3.8	2	3.3	1	5.5
Carboplatin/etoposide (n)	4	5.1	2	3.3	2	11.1
Busulfan/melphalan (n)	1	1.3	1	1.6	0	0
PEI (n)	1	1.3	1	1.6	0	0

^*^B-NHL include PCNSL (*n* = 10), DLBCL (*n* = 9), MCL (*n* = 4), FL (*n* = 3), and PMBCL (*n* = 1)

Legend: Survivors, patients who survived the ICU stay; non-survivors, patients who died during the ICU stay; *HDCT*, high-dose chemotherapy; *ASCT*, autologous stem cell transplantation; *HL*, Hodgkin lymphoma; *B-NHL*, B-cell non-Hodgkin lymphoma; *T-NHL*, T-cell non-Hodgkin lymphoma; *PCD*, plasma cell disorder; *HCT-CI*, Hematopoietic Cell Transplantation-specific Comorbidity Index; *CR*, complete remission; *PR*, partial remission; *SD*, stable disease; *PD*, progressive disease; *BEAM*, BCNU, etoposide, cytarabine, melphalan; *PEI*, cisplatin, etoposide, ifosfamide; *ICU*, intensive care unit; *PCNSL*, primary central nervous system lymphoma; *DLBCL*, diffuse large B-cell lymphoma; *MCL*, mantle cell lymphoma; *FL*, follicular lymphoma; *PMBCL*, primary mediastinal B-cell lymphoma

### Characteristics of ICU admission and procedures on the ICU

The median time interval between ASCT and admission to the ICU was 7 days (range: day 5–day 20). The most frequent causes for ICU admission were sepsis (53/79 patients, 67.9%) and neurological symptoms (9/79 patients, 11.4%) (Table 2). Severe neutropenia (defined as neutrophil count < 500/µl) at ICU admission was documented for 57/79 patients (72.2%; data not shown). The median SOFA score was 7 (range: 1–18). Bacteria were detected in 47/79 patients (59.5%) and viruses in 31/79 patients (39.2%); 11/79 patients (13.9%) had fungal infections (data not shown). During the stay on the ICU, 23/79 patients (29.1%) required mechanical ventilation (MV), 4/79 patients (5.1%) underwent renal replacement therapy (RRT), and the use of vasopressors was necessary in 35/79 patients (44.3%). Cardiopulmonary resuscitation (CPR) was performed in 9/79 patients (11.4%). The median duration of stay on the ICU was 5 days (range: 1–62 days) (Table 2).

**Table 2:** ICU characteristics of patients admitted to the ICU between the initiation of high-dose chemotherapy and day 30 after ASCT

	All patients		Survivors		Non-survivors	
		%		%		%
Total patients (n)	79		61	77.2	18	22.8
Major cause for ICU admission						
Sepsis (n)	53	67.9	41	67.2	12	66.7
Neurological symptoms (n)	9	11.4	8	13.1	1	5.6
Bleeding (n)	3	3.9	3	4.9	0	0
Arrhythmia (n)	2	2.6	2	3.3	0	0
Others (n) *	12	15.2	7	11.5	5	27.8
ICU admission characteristics						
Time from ASCT to ICU admission (days)—median (range)	7 (− 5–20)		7 (− 5–20)		7.5 (− 4–13)	
SOFA score at ICU admission—median (range)	7 (1–18)		7 (1–15)		9 (3–18)	
Lactate (mmol/l) at ICU admission—median (range)	1.3 (0.4–7.03)		1.2 (0.4–7.03)		2.1 (0.5–4.6)	
Procedures on the ICU						
MV (n)	23	29.1	8	13.1	15	83.3
NIV (n)	9	11.4	0	0	9	50
HFNO (n)	17	21.5	8	13.1	9	50
vvECMO (n)	2	2.5	0	0	2	11.1
RRT (n)	4	5.1	0	0	4	22.2
Vasopressors (n)	35	44.3	19	31.1	16	88.9
CPR (n)	9	11.4	2	3.3	7	38.9
ROSC (n)	8		2		6	
Survived (n)	2		2		0	
Duration of ICU stay (days)—median (range)	5 (1–62)		5 (1–59)		11 (1–62)	

^*^Others include respiratory failure for reasons other than pneumonia, metabolic disorders, drug intoxication or overdose, PD, cardiopulmonary resuscitation, and ileus

Legend: Survivors, patients who survived the ICU stay; non-survivors, patients who died during the ICU stay; *ICU*, intensive care unit; *ASCT*, autologous stem cell transplantation; *SOFA*, Sequential Organ Failure Assessment; *MV*, mechanical ventilation; *NIV*, non-invasive ventilation; *HFNO*, high-flow nasal cannula oxygen; *ECMO*, extracorporeal membrane oxygenation; *RRT*, renal replacement therapy; *CPR*, cardiopulmonary resuscitation; *ROSC*, return of spontaneous circulation; *PD*, progressive disease

### Outcome and risk factors

**Fig. 1:**
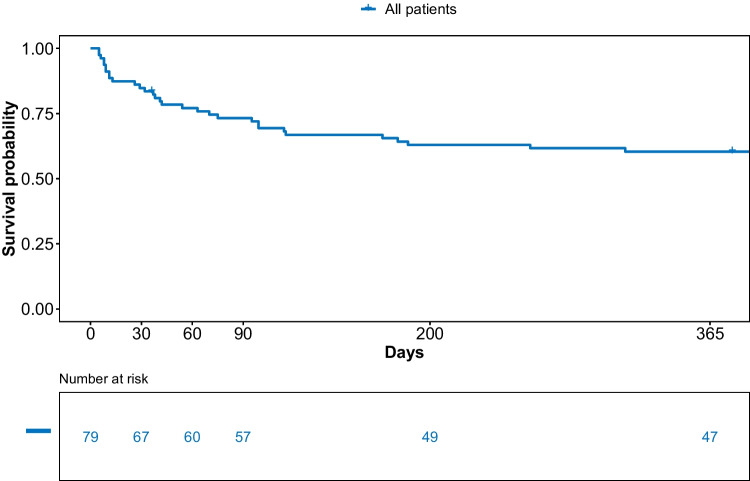
Overall survival of patients included in the analysis

The median observation time was 490 days (range: 5–2260 days) for all patients and 831 days (range: 36–2260 days) for surviving patients. Median OS was not reached. The ICU, hospital, 90-day, and 1-year survival rates were 77.2%, 77.2%, 72.2%, and 60.3%, respectively (Table 3, Fig. 1). Hence, all ICU survivors were also discharged from the hospital.

**Table 3:** Survival characteristics and causes of death among patients admitted to the ICU between the initiation of high-dose chemotherapy and day 30 after ASCT

		**%**
ICU survival (n)	61/79	77.2
Hospital survival (n)	61/79	77.2
90-day survival (n)	57/79	72.2
1-year survival (n)	47/78	60.3
Follow-up (days)—median (range) (all patients)	490 (5–2260)	
Follow-up (days)—median (range) (survivors)	831 (36–2260)	
Time from ICU admission to death (days)—median (range)	46 (1–2176)	
Time from ASCT to death (days)—median (range)	54 (5–2183)	
Cause of death		
Sepsis (n)	14/36	38.9
PD (n)	16/36	44.4
Others (n)	6/36	16.7

Legend: *ICU*, intensive care unit; *ASCT*, autologous stem cell transplantation; *PD*, progressive disease

Overall, 36 patients taken into account for the present analysis died during observation. The median time interval between ICU admission and death was 46 days (range: 1–2176 days). The median time interval between ASCT and death was 54 days (range: 5–2183 days). The most common causes of death were progressive disease (PD) (16/36 patients, 44.4%) and sepsis (14/36 patients, 38.9%) (Table 3). Deaths occurred during the stay on the ICU in 18/36 cases (50%). Of these, the majority were due to sepsis (13/18 patients; 72.2%; data not shown).

Patients with an HCT-CI score > 2 did not have a worse OS than patients with an HCT-CT score ≤ 2 (*p* = 0.25) (Supplemental Fig. 1A). In contrast, patients with stable disease (SD) or PD prior to high-dose chemotherapy (*p* = 0.0028) and those who required MV during the stay on the ICU (*p* < 0.0001) had a significantly reduced OS (Supplemental Fig. 1B, Supplemental Fig. 1C). Only 5/23 patients (21.7%) necessitating MV were alive at the last follow-up (Supplemental Fig. 1C).

According to a univariable analysis, patients with SD/PD prior to high-dose chemotherapy had a higher probability of death in comparison with patients with CR/PR (HR: 4.65, 95% CI: 1.43–17.04, *p* = 0.005). Patients requiring MV during their stay on the ICU were at an increased risk of death as compared with patients not necessitating MV (HR: 8.01, 95% CI: 2.38–32.33, *p* < 0.001). Higher age was also a poor-risk factor in terms of OS (HR: 1.04, 95% CI: 1.00–1.08 per year, *p* = 0.046) (Table 4).

**Table 4:** Univariable analysis (upper) and multivariable analysis (lower) of possible risk factors affecting survival

Variable	HR	95% CI	*p*
Univariable analysis			
SD/PD	4.65	1.43–17.04	0.005
MV	8.01	2.38–32.33	< 0.001
Age (per year)	1.04	1.00–1.08	0.046
Multivariable analysis			
SD/PD	1.2	0.49–2.92	0.692
MV	4.58	1.53–13.64	0.006
Age (per year)	1.02	0.99–1.05	0.146
HCT-CI (per point)	1.01	0.83–1.23	0.908
SOFA score at ICU admission (per point)	1.03	0.90–1.19	0.675

Legend: *SD*, stable disease; *PD*, progressive disease; *MV*, mechanical ventilation; *HR*, hazard ratio; *CI*, confidence interval; *HCT-CI*, Hematopoietic Cell Transplantation-specific Comorbidity Index; *SOFA*, Sequential Organ Failure Assessment

A multivariable Cox-regression analysis including the variables SD/PD prior to high-dose chemotherapy, MV, age, HCT-CI score at initiation of high-dose chemotherapy, and SOFA score at ICU admission was conducted. The analysis revealed MV as a risk factor with respect to OS (HR: 4.58, 95% CI: 1.53–13.64, *p* = 0.006). In contrast, SD/PD (HR: 1.20, 95% CI: 0.49–2.92, *p* = 0.692), age (HR: 1.02, 95% CI: 0.99–1.05 per year, *p* = 0.146), HCT-CI score at the initiation of high-dose chemotherapy (HR: 1.01, 95% CI: 0.83–1.23 per point, *p* = 0.908), and SOFA score at ICU admission (HR: 1.03, 95% CI: 0.90–1.19 per point, *p* = 0.675) were not associated with an increased risk of death (Table 4).

## Discussion

Data on patients admitted to the ICU during hospitalization for high-dose chemotherapy and ASCT are scarce. We therefore performed a single-center retrospective analysis comprising 79 individuals who had treatment on the ICU between the initiation of high-dose chemotherapy and day 30 after ASCT. The major findings were as follows: (1) 10.7% of patients who underwent high-dose chemotherapy and ASCT were admitted to the ICU within the first 30 days from ASCT; (2) outcome of patients included in the analysis was generally favorable with a 1-year OS of 60.3%; and (3) patients with SD/PD prior to high-dose chemotherapy and individuals who required MV during their stay on the ICU had an increased death rate.

In the present analysis, 10.7% of patients who had high-dose chemotherapy and ASCT between 2014 and 2020 were admitted to the ICU between the initiation of high-dose chemotherapy and day 30 after ASCT. The median age was 57 years, and females accounted for 38% of cases. Hence, the ICU admission rate was higher than in previous analyses. An analysis from a single institution in Germany comprising patients who underwent high-dose chemotherapy and ASCT between 2008 and 2014 indicated an ICU admission rate of 5.1%. According to an older retrospective study from Canada including patients treated with high-dose chemotherapy and ASCT between 2001 and 2006, the ICU admission rate was 3.3%. The median age of patients included in the present analysis was comparable to the previous reports (64 years and 57 years, respectively), whereas the proportion of females was lower than in the earlier studies (47% and 53%, respectively) [7, 8]. The higher ICU admission rate in the present analysis may at least in part be due to the more recent advent of data suggesting an improved OS for critically ill patients with hematologic malignancies who were admitted to the ICU early [12].

The most common cause for ICU admission in the present analysis was sepsis (53/79 patients, 67.9%). The median SOFA score at admission to the ICU was 7. These results are in line with an earlier report from Germany indicating that sepsis was the cause for ICU admission in 67% of cases; the median SOFA score at admission to the ICU was 8 [7]. In contrast, analyses addressing the outcome of individuals admitted to the ICU early after allogeneic stem cell transplantation revealed higher median SOFA scores up to 14 reflecting more severe illness in these patients [13–15].

In the present analysis, 23/79 patients (29.1%) required MV, 4/79 patients (5.1%) had RRT, and 35/79 patients (44.3%) needed vasopressors. A retrospective study from Brazil including 301 patients who had been treated with high-dose chemotherapy followed by ASCT for a hematologic malignancy and were admitted to the ICU within 1 year from ASCT indicated similar rates for MV and vasopressor use (29.9% and 35.5%, respectively) and a slightly higher rate for RRT (17.3%) [9]. Critically ill patients who had undergone allogeneic stem cell transplantation were reported to have a significantly higher need for MV, RRT, and vasopressors than individuals included in the present analysis. According to a retrospective study comprising 70 patients who were admitted to the ICU between the beginning of conditioning therapy and day 30 after allogeneic stem cell transplantation, MV, RRT, and vasopressors were necessary in 55.7%, 27.1%, and 64.3% of cases, respectively [13]. A registry-based analysis from Denmark investigating characteristics and outcomes of patients who had been admitted to the ICU within 3 years from the diagnosis of acute myeloid leukemia (AML) also reported higher rates for MV and RRT than observed in the present analysis. Within the time interval from 2013 to 2016, MV and RRT were required in roughly 40% and 20% of critically ill AML patients, respectively [16].

According to the present analysis, survival rates for patients admitted to the ICU during hospitalization for high-dose chemotherapy and ASCT were better than those previously reported for patients admitted to the ICU early after allogeneic stem cell transplantation and for critically ill patients with AML. The ICU and 1-year survival rates for patients included in the present analysis were 77.2% and 60.3%, respectively, whereas 3 analyses comprising patients admitted to the ICU during hospitalization for allogeneic stem cell transplantation indicated ICU survival rates ranging between 48.6 and 64.6% and 1-year survival rates ranging between 16.2 and 33% [13, 15, 17]. An older analysis evaluating the outcome of critically ill AML patients demonstrated an ICU survival rate of 45% [18]. These differences in favor of the patients from the present analysis who had undergone high-dose chemotherapy and ASCT are possibly attributable to the more transient immunosuppression in comparison with individuals who had allogeneic stem cell transplantation and the lower proportion of patients with a relevant activity of the underlying malignancy in comparison with individuals with AML admitted to the ICU.

Among patients included in the present analysis, insufficient response to treatment prior to high-dose chemotherapy and ASCT and the necessity of MV were associated with an impaired OS. This result does not come unexpected since it has been demonstrated for a multitude of malignancies that patients who do not achieve a remission upon treatment prior to high-dose chemotherapy and ASCT have a poor prognosis [19–21]. The necessity of MV represents a strong risk factor for a decreased survival in critically ill patients irrespective of the underlying disease [22, 23].

The present study has some limitations due to its single-center retrospective design. Unfortunately, it was also not possible to draw valid conclusions with regard to the question of whether outcomes differ between patients with different malignancies since the respective subgroups were too small. The inability to obtain sufficient data on the outcome of patients who had undergone high-dose chemotherapy and ASCT at our institution and were not admitted to the ICU represents an additional weakness since a comparison between individuals admitted to the ICU during hospitalization for high-dose chemotherapy and ASCT and patients not necessitating treatment on the ICU could thus not be conducted.

Taken together, the present study demonstrated that patients admitted to the ICU between the initiation of high-dose chemotherapy and day 30 after ASCT have a favorable overall outcome. Therefore, these patients should not be denied admission and treatment on the ICU.

## Supplementary information

Below is the link to the electronic supplementary material.

Supplementary file1 (PDF 55 KB)

## Data Availability

The data generated and analyzed are available upon request. Decisions in terms of data sharing will be made on a case-by-case basis.
