# Supplementary figures and images for: Characteristics and outcomes of patients undergoing high-dose chemotherapy and autologous stem cell transplantation admitted to the intensive care unit: a single-center retrospective analysis
Source: Ann Hematol. 2022 Nov 17;102(1):191–7. doi: 10.1007/s00277-022-05028-x (PMC9807528; doi:10.1007/s00277-022-05028-x)

# Supplemental Figure 1

## A

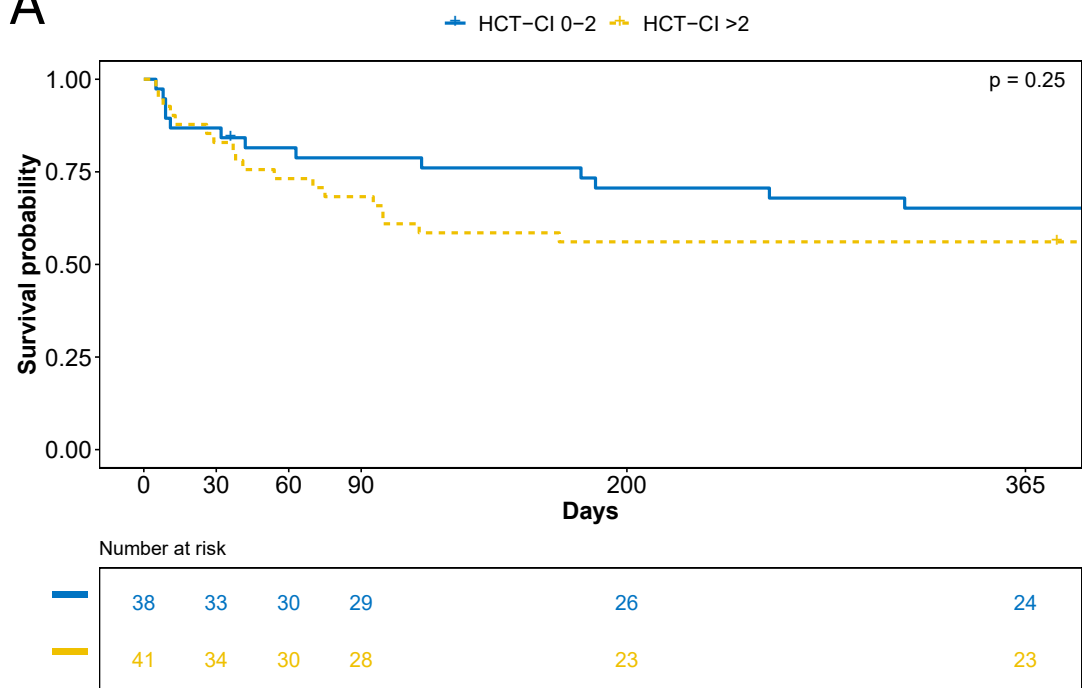

## B

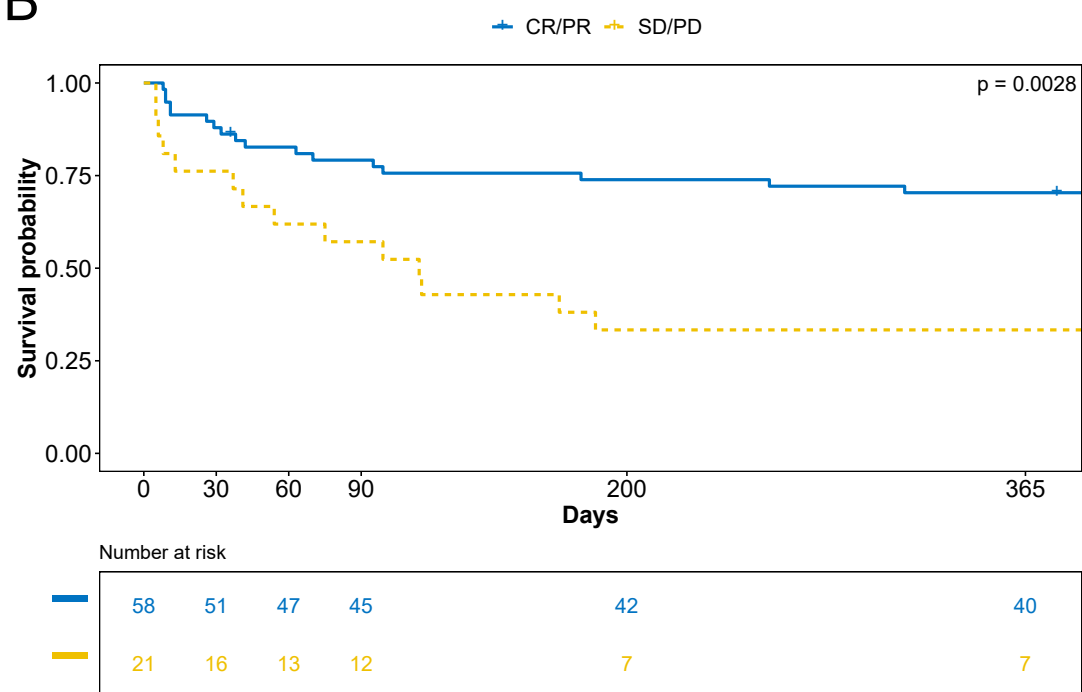

## C

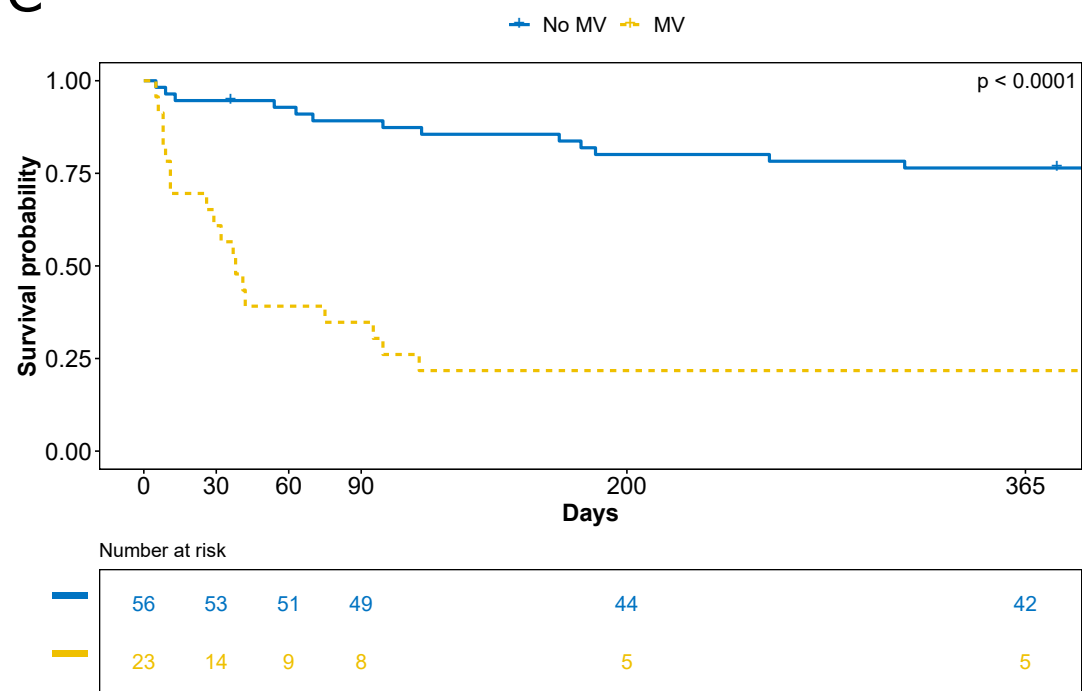

Supplement: Supplementary file 1 — Supplementary file1 (PDF 55 KB) [file 277_2022_5028_MOESM1_ESM.pdf]
